# Fast, accurate, and lightweight analysis of BS-treated reads with ERNE 2

**DOI:** 10.1186/s12859-016-0910-3

**Published:** 2016-03-02

**Authors:** Nicola Prezza, Francesco Vezzi, Max Käller, Alberto Policriti

**Affiliations:** Department of Mathematics and Informatics, University of Udine, via delle Scienze, Udine, 33100 Italy; Institute of Applied Genomics, via J. Linussio, Udine, 33100 Italy; Science for Life Laboratory, Tomtebodavägen 23A, Solna, 17165 Sweden

**Keywords:** Bisulfite, DNA methylation, NGS, Succinct hashing, BWT

## Abstract

**Background:**

Bisulfite treatment of DNA followed by sequencing (BS-seq) has become a standard technique in epigenetic studies, providing researchers with tools for generating single-base resolution maps of whole methylomes. Aligning bisulfite-treated reads, however, is a computationally difficult task: bisulfite treatment decreases the (lexical) complexity of low-methylated genomic regions, and C-to-T mismatches may reflect cytosine unmethylation rather than SNPs or sequencing errors. Further challenges arise both during and after the alignment phase: data structures used by the aligner should be fast and should fit into main memory, and the methylation-caller output should be somehow compressed, due to its significant size.

**Methods:**

As far as data structures employed to align bisulfite-treated reads are concerned, solutions proposed in the literature can be roughly grouped into two main categories: those storing pointers at each text position (e.g. hash tables, suffix trees/arrays), and those using the information-theoretic minimum number of bits (e.g. FM indexes and compressed suffix arrays). The former are fast and memory consuming. The latter are much slower and light. In this paper, we try to close this gap proposing a data structure for aligning bisulfite-treated reads which is at the same time fast, light, and very accurate. We reach this objective by combining a recent theoretical result on succinct hashing with a bisulfite-aware hash function. Furthermore, the new versions of the tools implementing our ideas|the aligner ERNE-BS5 2 and the caller ERNE-METH 2|have been extended with increased downstream compatibility (EPP/Bismark cov output formats), output compression, and support for target enrichment protocols.

**Results:**

Experimental results on public and simulated WGBS libraries show that our algorithmic solution is a competitive tradeoff between hash-based and BWT-based indexes, being as fast and accurate as the former, and as memory-efficient as the latter.

**Conclusions:**

The new functionalities of our bisulfite aligner and caller make it a fast and memory efficient tool, useful to analyze big datasets with little computational resources, to easily process target enrichment data, and produce statistics such as protocol efficiency and coverage as a function of the distance from target regions.

**Electronic supplementary material:**

The online version of this article (doi:10.1186/s12859-016-0910-3) contains supplementary material, which is available to authorized users.

## Background

DNA methylation is one of the most important epigenetic mechanisms, deeply affecting chromatin structure and gene expression. This modification consists of the addition of a methyl group to the fifth carbon in cytosine nucleotides, and can be detected to various degrees of resolution by using techniques such as *bisulfite sequencing* [1] (BS-seq), *microarrays* [2], or *methylated DNA immunoprecipitation* (MeDIP) [3] (to name a few). BS-seq is, up to date, the gold-standard technique to detect cytosine methylation at single-base resolution. By treating DNA with sodium bisulfite, unmethylated cytosines are converted to uracils, while methylated cytosines remain unaffected. After PCR amplification and sequencing, the overall effect is that reads contain thymines in places where the original genome contains unmethylated cytosines. Aligning BS-treated reads poses several computational challenges. First of all, reads coming from highly unmethylated genomic regions are characterized by low cytosine contents (since most of the Cs are converted into Ts). This loss of genomic complexity results in a higher number, with respect to more methylated regions, of ambiguous alignments in such regions, thus leading to potential biases. A second problem is the high number of C-to-T (genome-to-read) mismatches, which must be efficiently managed during the alignment phase and, most importantly, must not be source of penalization since they can potentially represent bisulfilte conversion rather than sequencing errors or SNPs. Moreover, space is often a concern during both alignment and methylation calling phases: the tools should use light data structures (fitting in main memory), and the methylation annotations—several data fields for each cytosine on both strands—should be somehow compressed on-the-fly by the caller itself.

These problems have been handled by existing tools to various degrees of efficiency. Software such as Bismark [4], BRAT-BW [5], BS-Seeker 2 [6], and MethylCoder [7] adopt the approach of converting both genome and reads files to a reduced alphabet, turning all Cs into Ts (and Gs into As while aligning the reversed reads), and calling a standard DNA aligner (typically either Bowtie/Bowtie 2 [8] or built-in) on the converted files. This technique removes the bias towards poorly methylated regions by leveling mapping efficiency across all regions, and solves the problem of efficiently dealing with the high number of C-to-T mismatches. A different approach—to allow C-to-T mismatches by using wild-card techniques—is adopted by tools such as BSMAP [9] and RMAP [10]. A mixture of the two strategies is also possible: the BS-aligner ERNE-BS5 (version 1) [11] uses a bisulfite-aware hash function invariant under bisulfite-induced mismaches, thus avoiding the conversion of genome and reads to a reduced alphabet.

As far as primary memory is concerned, the most memory-efficient aligners are those based on the Full-text in Minute space index (FM-index) [12] data structure (Bowtie 2, BWA-MEM [13], and SOAP 2 [14] to cite a few). Bismark, BS-seeker, and MethylCoder run instances of Bowtie on the converted files, and use the output alignments to infer methylation status of covered cytosines. ERNE-BS5 (version 1) and BSMAP, on the other hand, are based on less memory-efficient data strucures (hash tables) and require up to 20 GB to index the Human Genome. In this paper we will firstly show how we turned the hash index of ERNE-BS5 into a much more space-efficient data structure, the *dB-hash* [15]. This is done by using a hash function with three properties: *Hamming-aware* (faster search), *bisulfite-aware* (allow C-to-T mismatches), and *de Bruijn* (small space). The new versions of our tools heavily optimize also RAM usage during methylation calling (by using compressed structures), and I/O disk space usage: fastq files can be provided to ERNE-BS5 2 directly in compressed format, and the methylation caller ERNE-METH 2 can automatically compress methylation annotations (which results, in practice, to a 10-fold reduction in disk space usage for the output files).

Finally, we will discuss how experimental techniques different than whole-genome bisulfite sequencing (WGBS) require specialized tools able to exploit additional information provided by the protocol. Examples of such techniques are Reduced Representation Bisulfite Sequencing (RRBS) and capture followed by BS-seq, which permit focusing the sequencing effort on a small amount of well-defined targets. As a result, only regions of interest are sequenced (deeper than in WGBS) and consequently reliably analysed. A well known example of the latter strategy is Agilent’s SureSelect Human Methyl-Seq kit, which enriches (via probes hybridization) for 84 million bases (3.7 M CpGs) distributed among CpG islands, promoters, and Cancer and tissue-specific differentially methylated regions (DMRs). From the computational point of view, the problem of *aligning* BS-treated reads coming from a target-enrichment experiment is not much different from the one of aligning WGBS reads: even in the former case the alignment should be performed against the whole genome in order to discard false positive alignments that fall outside target regions (mainly due to out-of-target fragments that partially hybridize with the designed probes). Target information can, however, be exploited in the next pipeline stages—quality filtering of bases, methylation calling, DMRs identification—in order to speed-up and enhance the quality of the analysis process (which can be limited to target regions only). In this paper we will show how we extended the functionalities of ERNE-METH to include the possibility of exploiting target information (provided as a bed file) during methylation reconstruction.

## Methods

This section is divided in three main parts. First of all, we describe how we solved the problem of combining hashing and (succinct) indexing by using hash functions with the property of being homomorphisms on de Bruijn graphs. This result has been presented in [15], and here we just report the main ideas in order to be as self-contained as possible. In the same sub-section we also review the definition of *Hamming-awareness* [16, 17], a property guaranteeing fast search of strings under Hamming distance. Then, we show a hash function satisfying both Hamming-aware and de Bruijn properties, while at the same time being invariant under C-to-T mismatches. This hash function stands at the core of our new BS-aligner. Finally, the last sub-section is devoted to the description of the new version of our methylation caller, which has been extended with target-oriented functionalities and optimized for better space usage.

### Definitions

Throughout this paper we will work with the alphabet *Σ*_*DNA*_={*A*,*C*,*G*,*T*,*N*,*$*} (with *N* and *$* being the undefined base and the contig end-marker, respectively). With *n*, *m*, and *w* we will denote the reference’s length, the pattern’s length, and the (fixed) bit-size of a computer memory-word (i.e. the number 2^*w*^−1 is assumed to be the largest integer to fit in a memory word), respectively. As hash functions we will use functions of the form *h*:*Σ*^*m*^→{0,1}^*w*^ mapping length-*mΣ*-strings to length-*w* bit-strings. If necessary, we will use the symbol ${~}^{m}_{w}{h}$ instead of *h* when we need to be clear on *h*’s domain and codomain sizes. Given a string *P*∈*Σ*^*m*^, the value *h*(*P*)∈{0,1}^*w*^ will be also dubbed the *fingerprint* of *P* (in {0,1}^*w*^). With *T*∈*Σ*^*n*^ we will denote the *text* that we want to index using our data structure. ${T^{j}_{i}}$ will denote *T*[*i*,…,*i*+*j*−1], i.e. the length-*j* prefix of the *i*-th suffix of *T*.

The symbol ⊕ represents the exclusive OR (XOR) bitwise operator.

*d*_*H*_(*x*,*y*) will denote the Hamming distance between *x*,*y*∈*Σ*^*m*^.

### The dB-hash data structure

Hash tables are data structures supporting very fast queries, but they require a significant amount of space to be stored and maintained in main memory during alignment. This is due to the fact that such structures must store one pointer for each text position, thus incurring in a *Θ*(*n* log*n*)-bits overhead in the space usage (in addition to the plain text). An example of a hash-based BS-aligner is the first version of ERNE-BS5 [11], which required 19 GB of memory to index the Human genome (3.2 Gbp). Tools based on the FM index [12], instead, result in extremely lightweight alignment data-structures, being able to index the Human genome in as little as 1.1 GB [18]. This space efficiency, however, comes at the price of a slowdown, when *inexact* alignment queries are performed. This inefficiency comes from the fact that Burrows-Wheeler transform (BWT) based indexes implicitly encode the lexicographic ordering of all text suffixes; this results in *different* text substrings—even at low Hamming distances—potentially very far away in lexicographic ordering. Hence, aligners based on BWT must check input reads in their full length against each potential reference match—a step that can be avoided with *exact* matching, providing a BWT-interval in output. Backtracking (Bowtie[8, 18], for example, uses this strategy) and split-read strategies (SOAP 2 [14] splits the read into *k*+1 fragments while allowing for *k* errors) are two commonly employed techniques used to solve the inexact pattern matching problem while using BWT-indexes. When combined with quality-based heuristics, such techniques are much faster than classic hash-based methods [8, 14, 18].

A careful modification in the ordering of the text suffixes could result in similar text substrings being clustered together, with the important byproduct of a faster approximate-search query processing. This strategy is at the core of the dB-hash data structure [15], which, basically, permits collapsing similar patterns into similar (much shorter) fingerprints while at the same time maintaining prefix equivalences between them. More formally:

#### Definition 1.

Let *Σ*={0,…,|*Σ*|−1}. We say that a function *h*:*Σ*^*m*^→{0,1}^*w*^ is a *de Bruijn* hash function if and only if, for every pair of strings *P*,*Q*∈*Σ*^*m*^

$$P_{1}^{m-1} = Q_{0}^{m-1} \Rightarrow h(P)_{1}^{w-1} = h(Q)_{0}^{w-1} $$

In [15] is proved that a de Bruijn hash function, as defined above, is simply a homomorphism on de Bruijn graphs (having as sets of nodes *Σ*^*m*^ and *Σ*^*w*^, respectively). A *global* version of Definition 1 can be given, referring to an entire text by concatenating fingerprints of all its length-*m* substrings:

#### Definition 2.

Given ${~}^{m}_{w}{h}:\Sigma ^{m} \rightarrow \{0,1\}^{w}$ de Bruijn hash function and *n*≥*m*, the hash value of ${~}_{n-m+w}^{\qquad \;\, n}{h}$ on *T*∈*Σ*^*n*^, is the unique string ${~}_{n-m+w}^{\qquad \;\, n}{h}(T) \in \{0,1\}^{n-m+w}$ such that:

$${~}_{n-m+w}^{\qquad\;\, n}{h}(T)_{i}^{w} = {~}^{m}_{w}{h}\left({T_{i}^{m}}\right), $$

for every 0≤*i*≤*n*−*m*.

**Fig. 1:**
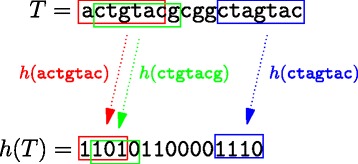
Example of a de Bruijn function *h* applied to a short DNA fragment. The main property characterizing such functions is that they preserve suffix-prefix overlaps

Since ${~}^{m}_{w}{h}$ univocally determines ${~}_{n-m+w}^{\qquad \;\, n}{h}$ and the two functions coincide on the common part *Σ*^*m*^ of their domain. In what follows we will simply use the symbol *h* to indicate both. Figure 1 should give an intuitive idea of the properties—formally introduced in Definitions 1 and 2—that characterize de Bruijn hash functions.

As a consequence of the above definitions we have that the hash value *h*(*T*) of the text retains enough information to support (probabilistic) pattern matching:

#### Lemma 1.

If *h* is a de Bruijn hash function, *n*≥*m*, and *P*∈*Σ*^*m*^ occurs in *T*∈*Σ*^*n*^ at position *i*, then *h*(*P*) occurs in *h*(*T*) at position *i*. The opposite implication does not (always) hold; we will refer to cases of the latter kind as false positives.

Lemma 1 proves that the text *T* augmented with a (succinct) index over *h*(*T*) can be used to perform pattern matching on *T*.

The second property we want in order to perform fast approximate pattern matching is *Hamming awareness* [16]:

#### Definition 3.

A hash function *h* is Hamming-aware if there exist

- a set $\mathcal {Z}(k) \subseteq \{0,1\}^{w}$ such that $|\mathcal {Z}(k)| \in \mathcal {O}\left (c^{k}w^{k}\right)$, for some constant *c*, and
- a binary operation *ϕ*:{0,1}^*w*^×{0,1}^*w*^→{0,1}^*w*^ computable in $\mathcal {O}(w)$ time,

such that if *P*∈*Σ*^*m*^ then the following inclusion holds:

(1) $$ \left\{h(P'):P'\in \Sigma^{m},\ d_{H}(P,P') \leq k\right\} \subseteq h(P)\ \phi\ \mathcal{Z}(k)  $$

**Fig. 2:**
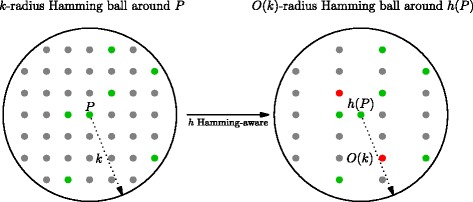
A Hamming-aware hash function collapses the *k*-radius Hamming ball centered on the pattern *P* to a $\mathcal {O}(k)$-radius Hamming ball centered on the hash *h*(*P*) of *P*. The latter sphere contains much less elements since |*h*(*P*)|≪|*P*|. In this picture, true positives (i.e. sequences that occur in the reference and whose hash values occur in the hash of the reference) are represented as green dots, true negatives (i.e. sequences that do not occur in the reference and whose hash values do not occur in the hash of the reference) are represented as gray dots, and false positives (i.e. sequences that do not occur in the reference and whose hash values occur in the hash of the reference) are represented as red dots. False negatives are not depicted here since they cannot be introduced by our hashing scheme

Intuitively, a Hamming-aware hash function *h* preserves Hamming distance information. This can be done while introducing a (provably small) number of false positives, as depicted in Fig. 2. The hash function we will use (described in detail in the next section) will satisfy this property. As a result, we can considerably reduce search space by only searching a Hamming ball centered around the hash value of the pattern. As shown in [15], given a hash function *h* satisfying both above introduced properties, we can build an index—called dB-hash—supporting approximate search queries in $\mathcal {O}((2\sigma)^{k}(\log n)^{k}\log m + (occ+1)\cdot m)$ time— *σ* being the alphabet size—while requiring only (2+*o*(1))*n* log*σ* bits of space. In [19] this technique is used to obtain a fast and sensitive short reads aligner. In the next section, we show how we can combine this strategy with the solution described in [11] in order to obtain a fast, sensitive, and lightweight bisulfite-treated reads aligner.

### A Hamming/bisulfite-aware de Bruijn hash function

A bisulfite-aware hash function satisfies the following property: the hash value *h*(*P*) of a pattern *P* is invariant under C-to-T substitutions. In order to use the same index for the forward and reverse genome, we furthermore require that the same property holds also for G-to-A substitutions. A straightforward way to accomplish this goal is to assign the same numeric encoding to Cs/Ts and Gs/As in the computation of *h*. This method is used in [11]; here we combine it with the two properties described in the previous section in order to obtain the hash function that will be used in our index.

First of all, we need to define an encoding $\alpha :\Sigma _{\textit {DNA}}\rightarrow \mathbb {N}$ for all letters in *Σ*_*DNA*_. For simplicity, we assume that reads and reference have been pre-processed so that all N and $ characters have been randomly converted to characters in {*A*,*C*,*G*,*T*} (this pre-processing is adopted also in practice). We assign the values *α*(*C*)=*α*(*T*)=0 and *α*(*G*)=*α*(*A*)=1. Let us denote with *α*(*P*), *P*∈{*A*,*C*,*G*,*T*}^∗^ the extension of *α* on strings, i.e. $\alpha (P)=\sum _{i=0}^{|P|-1} \alpha (P[\!i])\cdot 2^{|P|-i-1}$. The hash function that we use in our work is the following:

#### Definition 4.

With *h*_*BS*⊕_:{*A*,*C*,*G*,*T*}^*m*^→{0,1}^*w*^, *w*≤*m* we denote the hash function defined as

$$h_{\textit{BS}\oplus}(P) = \left(\bigoplus_{i=0}^{\lceil m/w\rceil-2}\alpha(P_{iw}^{w})\right) \oplus \alpha\left(P_{m-w}^{w}\right) $$

**Fig. 3:**
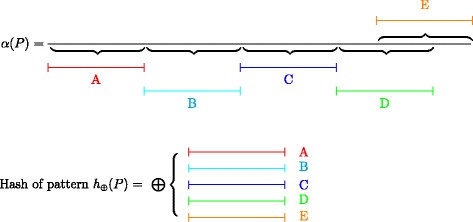
In the computation of *h*_*BS*⊕_(*P*), the bit-string *α*(*P*) is split in length-*w* blocks. All blocks except the last are non-overlapping and are taken from the beginning of *α*(*P*). The last block is taken from the end of *α*(*P*) and may overlap with the one preceding it. After this subdivision, all blocks are XOR-ed together, obtaining the fingerprint of *P*

Figure 3 gives a visual representation of the function described in Definition 4.

Using arguments from [15], it can be easily shown that *h*_*BS*⊕_ is a de Bruijn and Hamming-aware hash function. In particular, if *P*_1_,*P*_2_∈{*A*,*C*,*G*,*T*}^*m*^ and *d*_*H*_(*P*_1_,*P*_2_)≤*k*, then *d*_*H*_(*h*_*BS*⊕_(*P*_1_),*h*_*BS*⊕_(*P*_2_))≤2*k*, where the value *d*_*H*_(*h*_*BS*⊕_(*P*_1_),*h*_*BS*⊕_(*P*_2_)) is the Hamming weight of the bit-string *h*_*BS*⊕_(*P*_1_)⊕*h*_*BS*⊕_(*P*_2_). As a result, only fingerprints in the 2*k*-radius Hamming ball of center *h*_*BS*⊕_(*P*) (*P* being the searched pattern) must be considered during search. Experiments confirm (see Results section) that this strategy considerably improves query times of state-of-the-art bisulfite aligners based on backtracking.

### Methylation call

During the methylation calling phase, the alignments given as output by the bisulfite-aligner are used in order to derive a methylation value for every (covered) cytosine. This can be done by counting the number of Cs and Ts aligned under every genomic C (Gs and As aligned under every genomic G, respectively, for the reverse strand) and computing the *methylation score* defined as *β*(*i*)=*#C*(*i*)/(*#C*(*i*)+*#T*(*i*)), *i* being the (covered) genomic position containing the C and *#C*(*i*),*#T*(*i*) being the number of Cs/Ts aligned in position *i*, respectively (for the reverse strand this value is *β*(*i*)=*#G*(*i*)/(*#G*(*i*)+*#A*(*i*))). Despite the apparent simplicity of this task, there are several challenges that a methylation caller must face. First of all, notice that the tool must store two counters for every C and G in the genome. Using two simple arrays of, say, 16-bits integers (since we cannot establish a priori an upper bound to the maximum coverage) results in 4*n* bytes of space usage, which are added to the *n* bytes of the text itself. This space usage is 16 GB for the Human genome. The former version of our caller ERNE-METH (version 1) [11] used this simple strategy, which resulted in fast methylation calls at the price of high RAM consumption. In contrast, Bismark’s caller bismark_methylation_extractor performs this step in external memory, using low amounts of RAM at the price of speed and disk space usage. Here we describe how we improved the data structuring in ERNE-METH, obtaining a lightweight and fast methylation caller.

First of all we reduce the space used by the structure, by not storing integer counters for As and Ts positions. To this end we maintain a succinct bit-vector of *n*+*o*(*n*) bits to mark with a ’1’ Cs and Gs on the genome. The bit-vector is implemented in such a way to support constant-time *rank* operations [20]. Hence, mapping genomic positions to rank-space (i.e. every position *i* containing a C/G is mapped to *rank*(*i*)), we—implicitly—store counters for Cs and Gs only. This limits the “waste” of space for As and Ts to the ’0’ marked bits in the bit-vector, almost halving space usage with respect to the trivial solution (i.e. two length-*n* arrays). In order to save further space, we compress the counters to a bit size close to the minimum number of bits required to store each integer value. We divide each of the 2 counter arrays in blocks of length *bl* (*bl*=256 in our implementation). We denote as *capacity* of a block the bit-length of each of its counters. The structure is initialized building all blocks with initial capacity 1. Each time a counter exceeds the capacity of its block, we re-build the block with capacity increased by one. Notice that, if *bl* is chosen to be greater than or equal to the read length, this strategy requires time linear in the size of the input (i.e. number of aligned nucleotides) since a counter can be incremented at most once per read (and thus a block is re-built at most once per read). This strategy considerably reduces space usage of the methylation calling phase: in our experiments on Human data, the compressed counters required approximately 2.9 GB of RAM (as opposed to 12.8 GB of the trivial solution). This space is added to that of the text stored in a 3-bits per base format: 1.2 GB on the Human genome. The price to pay for this memory efficiency is a slight slowdown of the analysis—in practice, 4 times slower than the trivial solution—, which however is still much faster than Bismark’s external memory solution (see Results section).

A second issue concerns output disk space usage: a bed file with one line for every C and G in the genome can require a multiple of the space of the genome itself. Other tools (e.g. Bismark) do not compress these files, and thus require huge amounts of disk space to be run. We solve this problem simply by compressing on-the-fly methylation annotations, reducing their size to, approximately, that of the fasta input genome (up to 10-fold compression). ERNE-METH 2 implements both *gzip* and *bzip2* compression algorithms.

All the techniques described up to now make the new version of our methylation caller an extremely fast and lightweight tool, being able to exploit modern compression techniques in order to optimize memory requirements (both RAM and disk space) without significantly sacrificing query times. Quality of the methylation calling process can, however, be further improved by exploiting additional information provided by the experimental protocol. Target-enrichment techniques provide an example of such an application: in these cases, reads are known to come from specific regions of the genome of interest. Some protocols, such as *SureSelect Human Methyl-Seq* (Agilent), provide these regions as a bed file which can be used during methylation calling. In view of such experimental advances, we extended our caller to accept as input also a bed file containing target regions. This information is used to compute various additional statistics including percentage of bases on target, mean coverage on target, and distribution of coverage as a function of the distance from targets (useful to assess the number of bases to which extend targets on flanking sides). Moreover, reads falling outside target regions can be automatically discarded, methylation annotations can be outputted only on target Cs (producing much smaller files), and targets can be side extended to account for tails of coverage. Our caller has been tested on data produced with the SureSelect Human Methyl-Seq kit at the Swedish National Center for Molecular Biosciences, Science for Life Laboratory (SciLifeLab), Stockholm, Sweden. The study involved deep bisulfite sequencing of four samples of Human tissue, with one sample being sequenced at much higher depth (110M read pairs, corresponding to an average ≈30x coverage of targeted cytosines) than the others. The new functionalities of ERNE-METH 2 were useful in the process of assessing the protocol precision (fraction of reads aligned on target) and estimating (by using the most deeply sequenced sample) the optimal depth of coverage at which additional samples had to be sequenced. To conclude, we added two new annotation formats—EPP and Bismark’s cov—to our caller. This feature improves the compatibility of our ERNE-BS5 2/ERNE-METH 2 pipeline with widely-used downstream analysis tools such as RnBeads [21].

## Results

We compared the performances of our tool with two of the most widely used bisulfite aligners: Bismark version 0.14.3 [4] combined with both Bowtie 1 [18] and Bowtie 2 [8], and BSMAP version 2.90 [9]. The memory usage of the old version of our aligner and caller is also reported in order to highlight the improvements achieved with the new version. Tests on a public BS-seq Human library were performed to assess memory usage, speed, and alignment efficiency (i.e. mapped reads) of the tools in a real-case scenario. Additional tests on a simulated Human dataset were performed to compare the precision—in terms of mapping accuracy—of the bisulfite aligners. Finally, we ran a test on a simulated high-coverage dataset (*Arabidopsis thaliana* genome, 24.6x coverage) in order to demonstrate the correctness of our methylation caller ERNE-METH 2.

All experiments were performed on a intel core i7 machine with 12 GB of RAM running Ubuntu 14.04 operating system. Since Bismark executes 2 parallel Bowtie threads (one per strand), we executed ERNE-BS5 2 and BSMAP enabling multithreading with options –threads 2 and -p 2, respectively. In this way, all the tested aligners were allowed 2 parallel threads during execution. The choice of running 2 threads for each aligner is also motivated by the fact that Bismark’s memory usage increases linearly with the number *t* of parallel processes specified by the user (i.e. 2*t* FM-indexes are kept in memory, where *t*≥1). The memory usage of the other tested tools, on the other hand, is independent of *t* (a single data structure is shared by all parallel threads).

See Additional files 1 and 2 for further details on implementation usage and commands and parameters used for the experiments, respectively.

*Memory footprint and I/O utilization*

The new index of ERNE-BS5 2 significantly improves its former (hash) data structure, reducing the memory footprint from 19 to 3.85 GB on the Human genome (3.2 Gbp). This value is close to that of Bowtie 1’s and Bowtie 2’s FM indexes, which require only 2.95 and 3.2 GB to hold their FM indexes in memory, respectively. However, since Bismark executes (on different threads) 2 parallel instances of Bowtie and one instance running the Bismark process itself, its overall RAM utilization is of 9.1 GB when using Bowtie 1 and 9.6 GB when using Bowtie 2 (two FM indexes and the reference genome), much higher than that of ERNE-BS5 2.

As far as the methylation calling phase is concerned, Bismark’s caller bismark_methylation_extractor requires 2 GB of RAM on the Human genome, as opposed to the 4.1 GB used by ERNE-METH 2. This difference is due to the fact that Bismark’s caller is an external algorithm, and its RAM efficiency is paid in terms of disk space—170 GB in our experiments on Human—and time—bismark_methylation_extractor was one order of magnitude slower than ERNE-METH 2, as shown in the next subsections. Moreover, Bismark’s caller does not compress methylation annotations and, depending on the number of covered cytosines, its output file can easily reach tens of GB in size. In contrast, ERNE-METH 2 does not create intermediate files on disk (the whole analysis is carried out in RAM) and can directly compress methylation annotations, thus being an extremely lightweight tool able to optimize at best RAM and disk space usage (the latter being limited to the small—4.2 GB on the Human genome—compressed methylation annotation file). BSMAP’s methylation caller—methratio.py—has a declared RAM consumption of approximately 26 GB on the Human genome (too high to be executed on our testing machine).

**Fig. 4:**
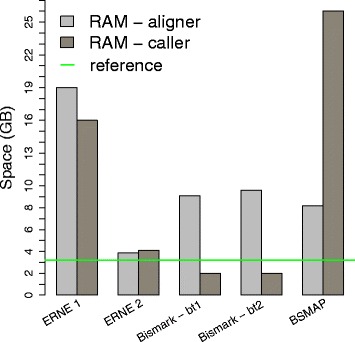
RAM requirements of ERNE 1, ERNE 2 (aligner: ERNE-BS5, caller: ERNE-METH), Bismark with Bowtie 1 (bt1), Bismark with Bowtie 2 (bt2) (aligner: bismark, caller: bismark_methylation_extractor), and BSMAP (aligner: bsmap, caller: methratio.py). This data has been collected from the real WGBS Human dataset experiment discussed in the next section, except for the memory usage of BSMAP’s methylation caller (for which we used the declared value of 26 GB on Human genome). The horizontal green line marks the space of the raw reference *fasta* file (3.2 GB)

We report in Fig. 4 the RAM space required by ERNE (versions 1 and 2), Bismark (with Bowtie 1 and 2), and BSMAP aligners and callers.

*Real WGBS dataset*

In order to compare the performances of ERNE-BS5 2, Bismark + Bowtie 1, Bismark + Bowtie 2, and BSMAP on a real dataset, we performed an experiment (alignment and methylation call) on the Human BS-seq library [22]. The dataset (lung adenocarcinoma cell lines) contains 50,544,402 pairs of 100 bp illumina (HiSeq 2500) reads. First of all, reads were quality-trimmed using ERNE-FILTER [23]. The resulting 48,706,207 pairs were subsequently aligned with ERNE-BS5 2, Bismark + Bowtie 1, Bismark + Bowtie 2, and BSMAP enabling multithreading on 2 cores for all the aligners as described at the beginning of this section. In this experiment we used default parameters for all the tools. After the alignment, we used ERNE-METH 2 and bismark_methylation_extractor to call methylation values. BSMAP’s methylation caller was excluded from this benchmark due to its high memory requirements (26 GB on the Human genome).

**Fig. 5:**
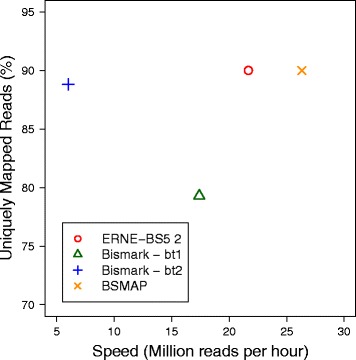
Speed and sensitivity of the tested bisulfite aligners. Hash-based aligners (BSMAP and ERNE-BS5 2) align the highest number of reads in unique positions and are faster than BWT-based aligners (Bowtie 1 and Bowtie 2). As expected, the hybrid ERNE’s dB-hash data structure is faster than BWT-based indexes and slower than classic hash-based indexes (BSMAP)

**Fig. 6:**
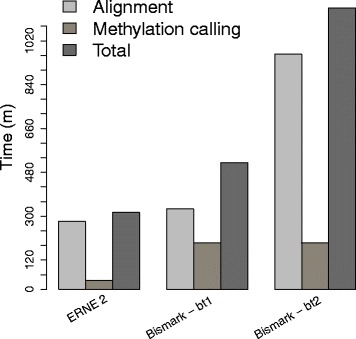
Time of alignment and methylation calling required by ERNE-BS 2 with ERNE-METH 2 and Bismark (Bowtie 1/Bowtie 2) with bismark_methylation_extractor. The ERNE 2 pipeline is from 1.6 to 3.6 times faster than the Bismark pipeline (using Bowtie 1/Bowtie 2, respectively). BSMAP is not shown here since its methylation caller required too much RAM (26 GB) to be run on our system

We report the results in Figs. 5 and 6. The plot in Fig. 5 shows throughput of the tools and fraction of reads aligned at unique positions (i.e. excluding unmapped reads and reads mapped in multiple positions). As expected, ERNE-BS5 2 exhibits the same mapping efficiency of the hash-based aligner BSMAP, and a speed which is intermediate between BWT-based (Bismark) and classic hash-based (BSMAP) aligners.

Figure 6 shows the time required by the tools to complete the whole alignment and methylation call pipeline on the dataset. ERNE 2 finished the analysis in considerably less time than Bismark + Bowtie 2 (being 3.6 times faster than Bismark), and in time comparable to that required by Bismark + Bowtie 1 (being 1.6 times faster than Bismark).

*Simulated WGBS datasets*

Real datasets cannot be used to test the accuracy of a bisulfite aligner/caller (in terms of number of correctly mapped reads / number of correctly called methylation values), since the underlying methylome is usually not known in advance. For this reason, the use of a simulated dataset is often a good choice if one wishes to assess such values. To compare the speed and accuracy of ERNE-BS5 2 with those of the other tested tools, we simulated a Human dataset (see below for details), aligned the reads, and measured speed and number of correctly mapped reads of all tools under different combinations of parameters. Finally, we tested the correctness of our caller ERNE-METH 2 by simulating a high-coverage dataset on the *Arabidopsis thaliana* genome and by comparing the predicted methylation values with the simulated ones.

**Datasets simulation** To generate the simulated datasets, we used custom scripts [24] and [25] in conjunction with the SimSeq reads simulator [26] to generate a directional [27] BS-seq dataset with simulated SNPs, indels, sequencing errors, (uniform) bisulfite conversions, and bisulfite conversion failures (usually, bisulfite protocols have a conversion efficiency around 98 *%*). See Additional file 2 for more details on the simulation procedure used.

**Mapping Accuracy** To measure mapping accuracy of the tested tools in terms of fraction of correctly aligned reads, we simulated (as described above) 13,850,280 read pairs from the build hg19 of the Human genome and aligned them with Bismark + Bowtie1, Bismark + Bowtie2, ERNE-BS5 2, and BSMAP on the reference. We evaluated only uniquely-mapping reads (multiple alignments are usually discarded before methylation reconstruction). A unique alignment was considered correct if and only if both chromosome and strand coincided with those outputted by SimSeq and if the alignment’s position was within 50 bases from the position outputted by Simseq (in order to account for indels and clipped bases).

As observed in [28], input data quality and alignment parameters can greatly influence aligner’s performances. In particular, the study reports that low sequencing error rates and trimmed data (see also [29]) improve mapping efficiency, while parameters such as number of mismatches allowed in the seed and number of mismatches allowed in the read are those having a major effect on the aligner’s speed and accuracy. In light of these observations, we decided to perform two types of comparisons. First of all, the tools were compared by using their default parameters (except for the multithreading options, as explained above, and disabling trimming since reads were already trimmed). This choice is motivated by the fact that default parameters are chosen by the software’s developers in order to offer a reasonably good trade-off between performances (memory/speed) and mapping accuracy, and therefore represent a good choice when one is interested in optimizing both these features. The default parameters used are: ERNE-BS52 (seed length in index construction: –bl 30, seed errors: –seed-errors 2, gaps enabled, threads: –threads 2, trimming disabled: –no-auto-trim), Bismark + Bowtie 1 (seed length: -l 28, seed errors: -n 1, gaps not supported, threads: -p 1), Bismark + Bowtie 2 (seed length: -L 20,bseed errors: -N 0, threads: -p 1), BSMAP (seed length: -s 16, only error-free seeds supported, mismatch rate: -v 0.08, gaps: -g 0, number of threads: -p 2).

**Fig. 7:**
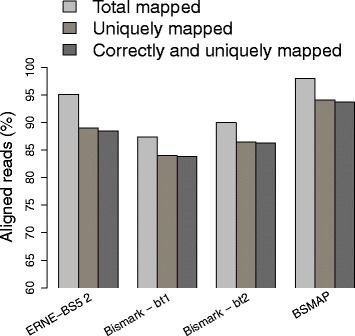
Simulated dataset, default parameters. Fraction of total mapped (multiple and unique), unique, and correctly mapped unique reads. In this experiment we used default parameters for all the tools

The results of this experiment are reported in Fig. 7. ERNE-BS5 2, Bismark + Bowtie 1, Bismark + Bowtie 2, and BSMAP map uniquely 89, 84, 86.6, and 94 % of the reads, respectively. All aligners map correctly most of the unique reads (>99 *%*). Memory usage and alignment speed of the tools were consistent with those reported in Figs. 4 and 5.

In order to compare the tools on a more common ground, we adopted the strategy of [28] and re-aligned the reads by setting the maximum number of allowed mismatches in the seeds to 0 for all the tools. Since Bismark does not permit to easily control the maximum number of allowed mismatches between the read and the reference, we allowed the tools to insert the maximum supported number of mismatches/indels in the remaining part of the alignment (i.e. seeds excluded). This is a reasonable choice since seeding is the most critical step of the alignment process; aligners usually implement in the seeding step most of their heuristics—e.g. backtracking (Bowtie) or hashing with mismatches (ERNE)—. In indel-free alignments, mismatches outside the seed are found simply by extension, a step that requires a good seeding strategy in order to be successfully performed.

The used parameters are: ERNE-BS52 (seed length in index construction: –bl 30, seed errors: –seed-errors 0, gaps enabled, threads: –threads 2, trimming disabled: –no-auto-trim), Bismark + Bowtie 1 (seed length: -l 28, seed errors: -n 0, gaps not supported, threads: -p 1), Bismark + Bowtie 2 (seed length: -L 20, seed errors: -N 0, threads: -p 1), BSMAP (seed length: -s 16, only error-free seeds supported, mismatch rate: -v 15, gaps: -g 3, number of threads: -p 2).

**Fig. 8:**
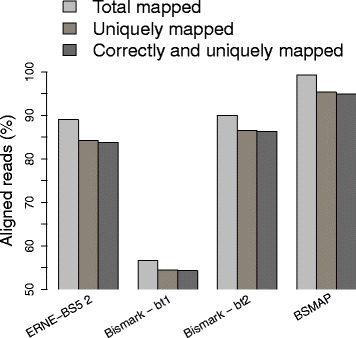
Simulated dataset, common parameters. Fraction of total mapped (multiple and unique), unique, and correctly mapped unique reads. In this experiment we disabled seed errors in all the tools, while tolerating the maximum allowed number of mismatches/indels in the rest of the read

The results of this experiment are reported in Fig. 8. ERNE-BS5 2, Bismark + Bowtie 1, Bismark + Bowtie 2, and BSMAP map uniquely 84.5, 54.5, 86.6, and 95.3 % of the reads, respectively. Again, all aligners correctly map most of the uniquely mapped reads.

The first noticeable effect of not allowing seed errors is that Bismark + Bowtie 1 has now very poor performances. This is due to the fact that Bowtie 1 uses a single-seed strategy and therefore requires seed errors in order to be reasonably efficient. All other tools use multiple seeding, and maintain overall a good alignment efficiency. The experiment shows that, under similar parameter combinations, ERNE-BS5 2 and Bismark + Bowtie 2 give very similar results. Despite both allowing 0 erros in the seed, in this experiment ERNE-BS5 2 and BSMAP align a different number of unique reads. This is due to the fact that BSMAP uses a much shorter seed than ERNE-BS5 2 (16 bp versus 30 bp), and is thus capable of aligning reads with more errors. BSMAP aligns slightly more reads than in the previous experiment since the only changed parameter is the allowed length of indels (the dafault is 0, while in this experiment was set to the maximum 3). However, allowing gaps in BSMAP heavily affects its running time: the tool required 9 h and 20 min to complete the alignment, for an overall throughput of 2.96 Million reads per hour. ERNE-BS5 completed the alignment in 1 hour and 36 min (17.3 Million reads per hour), Bismark + Bowtie 1 in 1 h and 20 min (20.77 Million reads per hour), and Bismark + Bowtie 2 in only 57 min (29.15 Million reads per hour).

**Methylation calling accuracy** Finally, we evaluated the accuracy of our methylation caller by simulating a high-coverage dataset on a medium-sized genome (*Arabidopsis thaliana*) and by comparing the results of ERNE-METH 2’s methylation calls to the simulated ground truth.

By using the protocol described at the beginning of this section, we generated a 24.6x coverage dataset on the Arabidopsis genome (60 M paired-end reads). Firstly, we aligned the reads with ERNE-BS5 2. After the alignment phase, we ran ERNE-METH 2 on the resulting bam file to generate the methylation profile. For the evaluation, we considered only Cs that had a coverage of at least 3, and we treated a C as methylated if its (called) methylation value was strictly greater than 0.5.

The whole pipeline took only 37 min to complete (27 min of alignment with ERNE-BS5 2 and 10 min of methylation call with ERNE-METH 2). 91 % of the reads were aligned uniquely by ERNE-BS5, and 95.85 % of all cytosines (on both strands) were covered by at least 3 reads. ERNE-METH 2 correctly called 99.9931 % of all covered cytosines’ methylation values.

## Discussion

In this paper, we described how to efficiently apply recent advances in succinct indexing techniques [15] to the bisulfite-alignment problem. To reach this goal, we employed a hash function with three properties: the first, *de Bruijn*, reduces space for the hash index from $\mathcal {O}(n\ \text {log}\ n)$ to (2+*o*(1))*n*log*σ* bits; experiments confirm that in practice this strategy reduces the size of the structure by one order of magnitude. The second property, *Hamming-awareness*, results in a considerable reduction in search space, thus speeding up (not only in theory but also in practice, as shown by our experiments) the alignment process. Finally, the *bisulfite-awareness* property enables efficient alignment of bisulfite-treated reads without significant penalization in query times with respect to standard DNA alignment (as demonstrated by the results here presented together with those reported in [19]). We implemented the bisulfite dB-hash data structure in the aligner ERNE-BS5 2, and showed that in practice it is competitive with state-of-the-art bisulfite aligners in terms of memory and speed, with comparable accuracy. On simulated reads, the hash-based aligner BSMAP was able to align reads more efficiently than ERNE-BS5 2 and Bismark, even though ERNE-BS5 2 was as efficient as BSMAP (and more than Bismark) on real data. Overall, ERNE-BS5 2 and ERNE-METH 2 showed to be extremely lightweight tools (much more than Bismark and BSMAP), offering a good tradeoff between memory, speed, and accuracy.

## Conclusions

The use of compressed data structures is fundamental in tasks where huge amounts of data need to be processed. By implementing recent theoretical results in this field of research, our tools are able to efficiently carry out in RAM both alignment and methylation calling processes using slightly more RAM space than the reference itself. In particular, our methylation caller ERNE-METH 2 is one order of magnitude faster than external memory algorithms performing the same task (e.g. Bismark’s caller), while at the same time using a relatively low amount of main memory. Other tools that perform this task completely in RAM (e.g. BSMAP’s methylation caller) require up to 6 times the space used by ERNE-METH 2.

Finally, the new target-oriented functionalities of ERNE-METH 2 make it a useful tool in target enrichment experiments.

## Availability

ERNE (*Extended Randomized Numerical alignEr*, version 2.1) is a short string alignment package whose goal is to provide an all-inclusive set of tools to handle short reads. ERNE comprises: ERNE-MAP, ERNE-DMAP, ERNE-FILTER, ERNE-VISUAL, ERNE-BS5, and ERNE-METH. ERNE is free software and distributed with an Open Source License (GPL V3) and can be downloaded at: http://ERNE.sourceforge.net.

## Additional files

Additional file 1
**Implementation usage.** (PDF 109 kb)

Additional file 2
**Commands used to perform the experiments.** (PDF 117 kb)

## Abbreviations

BS: bisulfite
BWT: burrows-wheeler transform
dB-hash: de Bruijn hash
EPP: epigenome processing pipeline applied at the broad institute
ERNE: extended randomized numerical alignEr
FM index: full text in minute space index
NGS: next-generation sequencing
PCR: polimerase chain reaction
RRBS: reduced representation bisulfite sequencing
SNP: single nucleotide polimorphism
WGBS: whole genome bisulfite sequencing
