# Supplementary material for: Fast, accurate, and lightweight analysis of BS-treated reads with ERNE 2
Source: BMC Bioinformatics. 2016 Mar 2;17(Suppl 4):69. doi: 10.1186/s12859-016-0910-3 (PMC4896272; doi:10.1186/s12859-016-0910-3)
Supplement: Additional file 1 — Implementation usage. (PDF 109 kb) [file 12859_2016_910_MOESM1_ESM.pdf]

Additional file 1 : implementation usage

Nicola Prezza[1], Francesco Vezzi[3], Max Käller[4], and Alberto Policriti[12]

[1] University of Udine, Department of Mathematics and Informatics, Udine, Italy
[2] Institute of applied genomics, Udine, Italy
[3] Science for Life Laboratory, Department of Biochemistry and Biophysics, Stockholm University, Solna, Sweden
[4] Science for Life Laboratory, KTH Royal Institute of Technology, Stockholm, Sweden

1 Implementation usage: ERNE

We implemented our algorithms and dats structures in the bisulfite aligner ERNE-BS5 and in the caller ERNE-METH, which are part of the short string alignment package ERNE 2 (`http://erne.sourceforge.net`).

1.1 Alignment

Briefly, the implementation needs only two commands to perform alignment of a set of bisulfite-treated reads (in fastq format) against a reference genome:

1. **Index construction**: To build the dB-hash index for bisulfite alignment call:

   ```
   erne-create --methyl-hash --fasta ref.fasta --reference-prefix idx
   ```

   where `ref.fasta` is the input fasta file and `idx` is the prefix of the output index (extension will be automatically added to obtain `idx.ebm`). We recommend to allocate at least $3.5n$ Bytes of RAM (where n is the reference length) while building the index. Building the Human genome index requires approximately 4 hours and 9.5GB of RAM on a intel core i7, 2.4GHz machine. After construction, the index will require approximately $1.2n$ Bytes on disk (and this will also be the RAM required for alignment).

2. **Alignment**: To align a pair of fastq files (containing bisulfite treated reads) to the indexed reference genome, just type

   ```
   erne-bs5 --reference idx.ebm --query1 q1.fq --query2 q2.fq --output ali.bam
   ```

   If reads are not paired, then specify just `--query1` parameter. erne-bs5 produces output in standard bam format. For more details, please read the manual at
   `http://erne.sourceforge.net/manual.php`.

1.2 Methylation call

Whole genome BS After alignment, only one command is needed to perform the methylation call step:

```
erne-meth --fasta ref.fasta --input ali.bam --output-prefix out --annotations-erne --compress
gz
```

The output files produced are:

– out_report.txt : a detailed human-readable report with the statistics about methylation and alignment.
– out_report_tabbed.txt : the same information as above, but in a more succinct and tabbed format (it can be used as input for a script in downstream analysis)
– out_erne_meth.txt.gz. A (gzip compressed) table in erne format displaying methylation levels for each cytosine in the reference. If methylation annotations in bismark cov or in EPP format are desired, specify also `--annotations-bismark` or `--annotations-epp`, respectively.

Target enrichment data The user can specify a bed file with the option `--target` containing the regions targeted by the protocol used. If this file is specified, erne-meth will compute additional statistics useful to assess the precision of the protocol. The following files are produced:

- out_on_target.txt : a table containing the percentage of on-target positions having coverage at least Ax, where $1 \leq A \leq 100$
- out_on_target.txt : a table containing the absolute number of out-of-target positions having coverage at least Ax, where $1 \leq A \leq 100$
- out_out_target_cov_distribution.txt : a table containing a set of triples $\langle D, C, N \rangle$. Each triple has the following meaning: there are N out-of-target positions having coverage C and such that the nearest target region is at distance D.

One common effect in target enrichment protocols are the coverage tails at the borders of the target regions. For this reason, it is useful to extend each target region by a number of bases on its extremities. Use the option –extend-target N to to extend each target region by N bp on its extremities.

Specify –on-target-annotations to print only on-target Cytosines in the files generated with options –annotations-epp and –annotations-bismark.
