# Supplementary material for: Fast, accurate, and lightweight analysis of BS-treated reads with ERNE 2
Source: BMC Bioinformatics. 2016 Mar 2;17(Suppl 4):69. doi: 10.1186/s12859-016-0910-3 (PMC4896272; doi:10.1186/s12859-016-0910-3)
Supplement: Additional file 2 — Commands used to perform the experiments. (PDF 117 kb) [file 12859_2016_910_MOESM2_ESM.pdf]

Additional file 2 : commands used to perform the experiments

Nicola Prezza[1], Francesco Vezzi[3], Max Käller[4], and Alberto Policriti[12]

[1] University of Udine, Department of Mathematics and Informatics, Udine, Italy
[2] Institute of applied genomics, Udine, Italy
[3] Science for Life Laboratory, Department of Biochemistry and Biophysics, Stockholm University, Solna, Sweden
[4] Science for Life Laboratory, KTH Royal Institute of Technology, Stockholm, Sweden

1 Creation of the indexes

Bismark + Bowtie 1

In the following, the variable `path_to_genome_folder` stores the path containing the original fasta file.

```
bismark_genome_preparation $path_to_genome_folder
```

Bismark + Bowtie 2

```
bismark_genome_preparation --bowtie2 $path_to_genome_folder
```

ERNE-BS5

```
erne-create --fasta genome.fasta --output-prefix ref --methyl-hash
```

BSMAP

BSMAP automatically builds his hash index before alignment.

2 Reads simulation

To simulate the reads, we used SimSeq (`https://github.com/jstjohn/SimSeq`) in combination to custom scripts that can be downloaded at `github.com/nicolaprezza/test-bs-aligner`. The main script of this pipeline is `pipeline_bismark_and_erne.sh`. The pipeline simulates a methylation experiment, executes the erne/bismark aligners and methylation callers, and counts the number of correctly called methylations and total number of called methylations. To modify parameters of the simulation (e.g. number of reads, error rate, ecc...), edit testcase-bs-aligner-default.sh. The script requires the following programs to be pre-installed:

– SimSeq (`github.com/jstjohn/SimSeq`) installed in ~/workspace/SimSeq/

– samtools (http://www.htslib.org/)
– fastx-mutate-tools (https://github.com/nicolaprezza/fastx-mutate-tools) installed in ~/workspace/fastx-mutate-tools/
– realpath
– fastx-toolkit (http://hannonlab.cshl.edu/fastx_toolkit/)
– gawk, awk
– erne (http://erne.sourceforge.net/)
– bismark (www.bioinformatics.babraham.ac.uk/projects/bismark/)

The workflow was designed as follows: firstly, we introduced 0.5% of SNPs at random positions in the input genome, producing a `snps.fasta` file. This file was further mutated generating two bisulfite-converted files, `C_to_T.fasta` and `G_to_A.fasta`, where we uniformly substituted with probability 0.5 Cs into Ts (methylations on forward strand) and Gs into As (methylations on reverse strand), respectively. Together with these two files, we generated a `bed` file containing the corresponding methylation values (0 or 1) for each cytosine in the genome.

Let N be the total number of simulated read pairs. We used SimSeq to simulate $0.02 \cdot N$ read pairs with sequencing errors using `snps.fasta` as reference file. SimSeq was executed using the built-in Illumina error model. These reads, representing BS-conversion failures, were saved in two files `query1.fastq` and `query2.fastq`. In order to generate bisulfite-converted reads, we used SimSeq to simulate further $0.98 \cdot N$ pairs from the reference `C_to_T.fasta` and $0.98 \cdot N$ pairs from the reference `G_to_A.fasta`, being careful to select from the fist batch only pairs having the first/second read on forward/reverse strand, respectively, and from the second batch only pairs having the first/second read on reverse/forward strand, respectively. Pairs extracted from the second batch had to be further swapped (i.e. we exchanged the role of read 1 and read 2 in each pair) in order to produce a valid directional dataset. All such simulated pairs were finally appended to the end of the files `query1.fastq` and `query2.fastq`. To conclude, we introduced indels in the files `query1.fastq` and `query2.fastq`, using 0.0003 and 0.8 as open and extend probability, respectively (this corresponds at inserting 3 indels every 10000 base pairs with average indel length equal to 5). After the simulation, `query1.fastq` and `query2.fastq` were quality-trimmed using ERNE-FILTER.

3 Read filtering

We used `erne-filter` to trim and quality-filter the real dataset. The command executed is:

```
erne-filter --query1 query1.fastq \
            --query2 query2.fastq \
            --output-prefix filtered
```

4 Alignments

4.1 Default parameters

In this section we don't write the parameters for which we used the default values. See the main text for more details on the default values.

ERNE-BS5 2 In the following, `ref.ebm` is the reference file created with `erne-create` (see above).

```
erne-bs5 --reference ref.ebm \
         --query1 query1.fastq \
         --query2 query2.fastq \
         --output alignment.bam \
         --threads 2 \
         --no-auto-trim
```

Bismark + Bowtie 1 In the following, the variable `path_to_genome_folder` stores the path containing the original fasta file. The variables `output_dir` and `temp_dir` store the path to the output directory and to a directory for the temporary files, respectively.

```
bismark $path_to_genome_folder \
        -1 query1.fastq \
        -2 query1.fastq \
        -o $output_dir \
        --temp_dir $temp_dir
```

Bismark + Bowtie 2 In the following, the variable `path_to_genome_folder` stores the path containing the original fasta file. The variables `output_dir` and `temp_dir` store the path to the output directory and to a directory for the temporary files, respectively.

```
bismark $path_to_genome_folder \
        --bowtie2 \
        -1 query1.fastq \
        -2 query1.fastq \
        -o $output_dir \
        --temp_dir $temp_dir
```

BSMAP

```
bsmap   -a query1.fastq \
        -b query1.fastq \
        -d genome.fasta \
        -o output.bam\
        -p 2
```

4.2 Common parameters (no seed errors)

In this section we don't write the parameters for which we used the default values. See the main text for more details on the default values.

ERNE-BS5 2 In the following, `ref.ebm` is the reference file created with `erne-create` (see above).

```
erne-bs5 --reference ref.ebm \
         --query1 query1.fastq \
         --query2 query2.fastq \
         --output alignment.bam \
         --threads 2 \
         --no-auto-trim \
         --seed-errors 0
```

Bismark + Bowtie 1 In the following, the variable `path_to_genome_folder` stores the path containing the original fasta file. The variables `output_dir` and `temp_dir` store the path to the output directory and to a directory for the temporary files, respectively.

```
bismark $path_to_genome_folder \
        -1 query1.fastq \
        -2 query1.fastq \
        -o $output_dir \
        --temp_dir $temp_dir  \
        -n 0
```

Bismark + Bowtie 2 In the following, the variable `path_to_genome_folder` stores the path containing the original fasta file. The variables `output_dir` and `temp_dir` store the path to the output directory and to a directory for the temporary files, respectively.

```
bismark $path_to_genome_folder \
        --bowtie2
        -1 query1.fastq \
        -2 query1.fastq \
        -o $output_dir \
        --temp_dir $temp_dir
```

BSMAP

```
bsmap   -a query1.fastq \
        -b query1.fastq \
        -d genome.fasta \
        -o output.bam\
        -p 2  \
        -g 3 \
        -v 15
```

5 Methylation call

Bismark

In the following, the variable path_to_genome_folder stores the path containing the original fasta file. The variable output_dir stores the path to the output directory. Notice that with option --gzip some of the output files are compressed, but the methylation annotations (.cov file) are output in an uncompressed format. File alignment.bam is the alignment output by bismark.

```
bismark_methylation_extractor -p \
                              --output $out_dir \
                              --gzip \
                              --genome_folder $path_to_genome_folder \
                              alignment.bam
```

ERNE-METH

In the following, alignment.bam is the alignment output by erne-bs5, and genome.fasta is the same genome used to build the index with erne-create. The command produces methylation annotations in erne format and statistics files, all having as prefix the content of the variable out_prefix. With the option --compress gz, erne-meth produces compressed methylation annotations.

```
erne-meth --input alignment.bam \
          --fasta genome.fasta \
          --annotations-erne \
          --output-prefix $out_prefix \
          --compress gz
```
